# Identification of novel variants in carbamoyl phosphate synthetase 1 gene and comparative pathogenicity assessments of CPS1 missense variants following ACMG/AMP-ClinGen recommendation for computational tools

**DOI:** 10.1016/j.ymgmr.2025.101208

**Published:** 2025-03-22

**Authors:** Fei Li, Qin Cai, Wei Ji, Miao Xu, Guoli Tian, Fanyi Zeng

**Affiliations:** a Shanghai Children's Hospital, Shanghai Institute of Medical Genetics, Shanghai Jiao Tong University School of Medicine, Shanghai 200040, China; b Department of Histo-Embryology, Genetics and Developmental Biology, Shanghai Jiao Tong University School of Medicine, Shanghai 200025, China; c Neonatal Screening Center, Children's Hospital of Shanghai, Shanghai Jiao Tong University, Shanghai 200040, China; d NHC Key Laboratory of Medical Embryogenesis and Developmental Molecular Biology & Shanghai Key Laboratory of Embryo and Reproduction Engineering, Shanghai 200040, China; e School of Pharmacy, Macau University of Science and Technology, Macau, China

**Keywords:** Carbamoyl phosphate synthetase I deficiency, Urea cycle disorder, Pathogenicity interpretation, Missense variant

## Abstract

Carbamoyl phosphate synthetase I (CPS1) deficiency is a rare autosomal recessive metabolic abnormality cause by dysfunctionality of CPS1 and often result in unfavorable outcome. In this study, we presented the detailed laboratory features and genetic analysis of two patients with heterozygous variants of CPS1, c.1927 A > G (p.Asn643Asp), c.2375 T > G (p.Met792Arg), c.3443 T > A (p.Met1148Lys) in patient 1; c.3784C > T (p.Arg1262Ter), c.3734 T > A (p.Leu1245His) in patient 2, respectively. c.1927 A > G (p.Asn643Asp) and c.2375 T > G (p.Met792Arg) are novel out of 5 variants and classified as variants of uncertain significance (VUS) under the guidelines of ACMG/AMP-ClinGen. Structure-based analysis of 4 missense variants indicates deleterious alterations to the protein. Since the employment of genetic testing as a clinical diagnostic tool, distinguishing pathogenic from polymorphic changes poses significant problems for geneticists. As recommendation for PP3/BP4, the computational tools for missense variant have been published, we performed a comparative evaluation for pathogenicity interpretation in our patients and in ClinVar database regarding CPS1 missense variants under the updated guidelines of ACMG/AMP-ClinGen. The application of computational tools under the ACMG/AMP-ClinGen criteria revealed an increased sensitivity for pathogenicity evaluation, from variants of uncertain significance (VUS) to likely pathogenic (LP) in previously reported cases; while for variants without clinic information in the ClinVar database, the pathogenicity assessment of VUS remained, and shows a more optimistic and reliable clinical application in molecular diagnosis.

## 1 Introduction

Carbamoyl phosphate synthetase I (CPS1) is one of six enzymes in urea cycle that catalyzes the conversion of ammonia and bicarbonate to carbamoyl phosphate. The homozygous or compound heterozygous variants in CPS1 gene causes this autosomal recessive (AR) inherited metabolic disorders, Carbamoyl phosphate synthetase I deficiency (CPS1D; MIM #237300). The exact incidence of CPS1D is unknown while the estimated incidence varies from 1:539,000 (Finland) [1], 1:800,000 (Japan) [2] to 1:1,300,000 (USA) [3]. Hyperammonemia is an indicative feature of Urea cycle disorders (UCDs), but it could not distinguish CPS1D and other UCDs [4]. Totally dysfunctional CPS1 results in neonatal onset of fatal hyperammonemia with nonspecific initial symptoms. In milder patients who own partially functional protein, CPS1D might be occurred at almost any time of life by multiple triggers [[5], [6], [7]].

Tandem mass spectrometry (MSMS) of dried blood spots (DBSs) is the most convenient, practical, and cost-effective way for newborn screening (NBS) and inherited metabolic disorders (IMDs) diagnosis for decades [[8], [9], [10]]. Given urea cycle is the sole source of endogenous production of arginine, ornithine, and citrulline, levels of these amino acid are indicators of UCDs, but the sensitivity and specificity of this method are not high enough for detection some types of UCDs [11]. Genetic testing in UCDs is strongly recommend to confirm the diagnosis, allow for genetic counseling and in some instances provide information on the disease course [12].

In 2015, the American College of Medical Genetics and Genomics (ACMG) and the Association for Molecular Pathology (AMP) published the guideline that provides a framework for sequence variant interpretation, combination of different criteria were provided to assign a pathogenicity assertion for certain sequence variant [13]. Some of these criteria have been updated several times since then [[14], [15], [16], [17]], but the assessments of missense variants are still a hurdle in front. The ClinGen recommendations for PP3/BP4 criteria were published [18], which might lead to great change in missense variants pathogenicity assertion.

Here we reported, assessed the pathogenicity of two CPS1D patients with 5 variants and performed stability, flexibility and structural analysis, made a comparation of the pathogenicity interpretation of CPS1 missense variant due to the updated recommendation for PP3/BP4 in our patients as well as in ClinVar database.

## 2 Materials and methods

### 2.1 Samples and ethical approval

Two dried blood spots samples of patients screened by MSMS are from Shanghai Children's Hospital in this study. This study was approved by Ethics Review Committee of Shanghai Children's Hospital, the Affiliated Children's Hospital of Shanghai Jiao Tong University School of Medicine in China.

### 2.2 Variant data sets

Missense variants of CPS1 that reviewed as “Uncertain significance” and “Conflicting interpretations of pathogenicity” in ClinVar database (Data up to July 13, 2023) were retrieved for comparative pathogenicity evaluation in this study. These variants were divided into reported and unreported groups based on whether they were available from the reliable literatures. Usually, the clinical information was provided more or less in reported variants group as they were published in case or cohort studies, but unreported variants group have no more information than variants.

### 2.3 MSMS

The level of amino acids level in blood was detected by MSMS with Quattro micro (Waters, QAB1828) and NeoBase Non-derivatized MSMS kit (PerkinElmer, 3040-0010Z), then analyzed by NeoLynx Browser software (Waters).

### 2.4 Genomic DNA extraction, next-generation sequencing, Bioinformatic analysis and sanger sequencing

Genomic DNA was extracted from the dried blood spots using a Nucleic acid extraction reagent (Uni-medica Technology Co., Ltd., Shenzhen, China) then was enriched for targeted exon regions of 130 genes (Table S1) which include all UCDs genes and sequencing was carried out using the Illumina NGS technology (Human Inherited Metabolic Diseases 130 Genes-Variant Assay, Uni-medica Technology Co., Ltd., Shenzhen, China). Bioinformatic Analysis are performed by Shenzhen Uni-medica Technology Co., Ltd. The obtained mean exome coverage was over 95 %, and average sequencing depth of each sample ≥ 100×. Qualified reads were aligned to the human reference genome (GRCh37/hg19) with Burrows-Wheeler Aligner. Annotation was carried out by ANNOVAR. The variants were compared with the established human CPS1 sequences, Matched Annotation from NCBI and EMBL-EBI (MANE) selected transcript NM_001875. Highly suspicious variants from patients with abnormal mass spectrometry results are confirmed by sanger sequencing by Shenzhen Uni-medica Technology Co., Ltd.

### 2.5 Genetic-phenotypic analysis and ACMG/AMP-ClinGen interpretation

Variants filtration was performed following two rules: (1) Allele frequency is less than 1 % MAF (minor allele frequency) in the population according to public database (gnomAD, http://gnomad.broadinstitute.org/); (2) Target genes were associated with the patients' phenotypes. In silico predictions for missense variants was carried out by REVEL, the ensemble predicting method based on a combination of scores from 13 individual tools [19]. Threshold scores for this meta-predictor tools have been defined ≥0.7 for PP3 (Multiple lines of computational evidence support a deleterious effect on the gene or gene product) and ≤0.4 for BP4 (Multiple lines of computational evidence suggest no impact on gene or gene product) due to ACGS Best Practice Guidelines for Variant Classification in Rare Disease 2020 [20]. The updating threshold scores according to in silico tool for PP3_Strong are defined≥0.932, PP3_ Moderate are defined between 0.773 and 0.932, PP3 are defined between 0.773 and 0.644 and threshold scores for BP4_VeryStrong are defined≤0.003, BP4_Strong are defined between 0.003 and 0.016, BP4_ Moderate are defined between 0.016 and 0.183, BP4 are defined between 0.183 and 0.290. All the selected variants were classified as pathogenic (P), likely pathogenic (LP), uncertain significance (VUS), likely benign (LB), or benign (B). All the potential genetic lesions detected were validated by sanger sequencing by Shenzhen Uni-medica Technology Co., Ltd.

The clinical significance of all the variants found in probands in this study and the missense variations in ClinVar Database (https://www.ncbi.nlm.nih.gov/clinvar/) are assessed following the ACMG/AMP-ClinGen guidelines. To evaluating the impact of PP3/BP4 criteria updating in missense variant pathogenicity interpretation, we made a comparative evaluation before and after the criteria application. The criteria are based on automated criteria in Varsome [21] then calibrated manually due to ACMG/AMP-ClinGen criteria. PM1 (Located in a mutational hot spot and/or critical and well-established functional domain without benign variation) are applied due to automated criteria in Varsome as to maintain a consistent algorithmic results. PS3 (Well-established in vitro or in vivo functional studies supportive of a damaging effect on the gene or gene product) are not applied in missense variants' assessment due to recommendations for application of the functional evidence criterion [16], mainly because the unclear thresholds for defining a functionally normal, indeterminate, or functionally abnormal result. Reported variant in cases are evaluated for PM3 criterion (For recessive disorders, detected in trans with a pathogenic variant) exclude cases without enough information [[22], [23], [24], [25], [26], [27], [28], [29], [30], [31], [32], [33], [34], [35], [36], [37], [38], [39], [40], [41], [42]]. Given CPS1D is a kind of metabolic disorder, PP4 (Patient's phenotype or family history is highly specific for a disease with a single genetic etiology) is widely used in variants that found in confirmed patients reported in references rather than in the group which have declared enzyme activity assay [23]. And PP4 is also applied in our patients as their phenotype as well as the genetic information are highly specific for UCDs. References for pathogenicity in table S2 have been listed separately by PMID.

### 2.6 Protein structure analysis

Changes in protein stability (ΔΔG, kcal/mol) of variants are predicted by PremPS [43], mCSM [44] and DynaMut2 [45], which are separately based on algorithms or models. Flexibility changes are predicted by PredyFlexy [46]. Dynamic changes in structure are provided by PyMOL. Structure-based stability and flexibility predictors are starting from the 5DOU and 5DOT structures. Alterations of function are annotated by HOPE [47]. 3D-structure of HOPE predictors are starting from PDB structures automatically.

## 3 Results

### 3.1 Clinic characteristics and genetic analysis of CPS1D patients

The laboratory data of 2 patients show abnormal metabolism of amino acids, implies urea cycle disorders. The amino acid metabolic results of mass spectrometry from the 2 patients are summarized in Table 1.

**Table 1:** Tandem mass spectrometry profile of the 2 patients with CPS1D.

	Patient 1(μmol/L)	Patient 2(μmol/L)	Reference value (μmol/L)
ARG	15.4	5.26	0.8–60
ARG/ORN	0.11	0.1	0.00–1.00
CIT	7.08	1.58 ↓	6.00–50.00
CIT/ARG	0.55	0.3	0.20–18.00
GLN	4325.3 ↑	1104.41	5.0–1150.0
ORN	113.6	54.43	35.00–360.00
ORN/CIT	16.14	34.43 ↑	1.60–25.00

Abbreviations: ARG, Arginine; ORN, Ornithine; CIT, Citrulline; GLN, Glutamine.

**Fig. 1:**
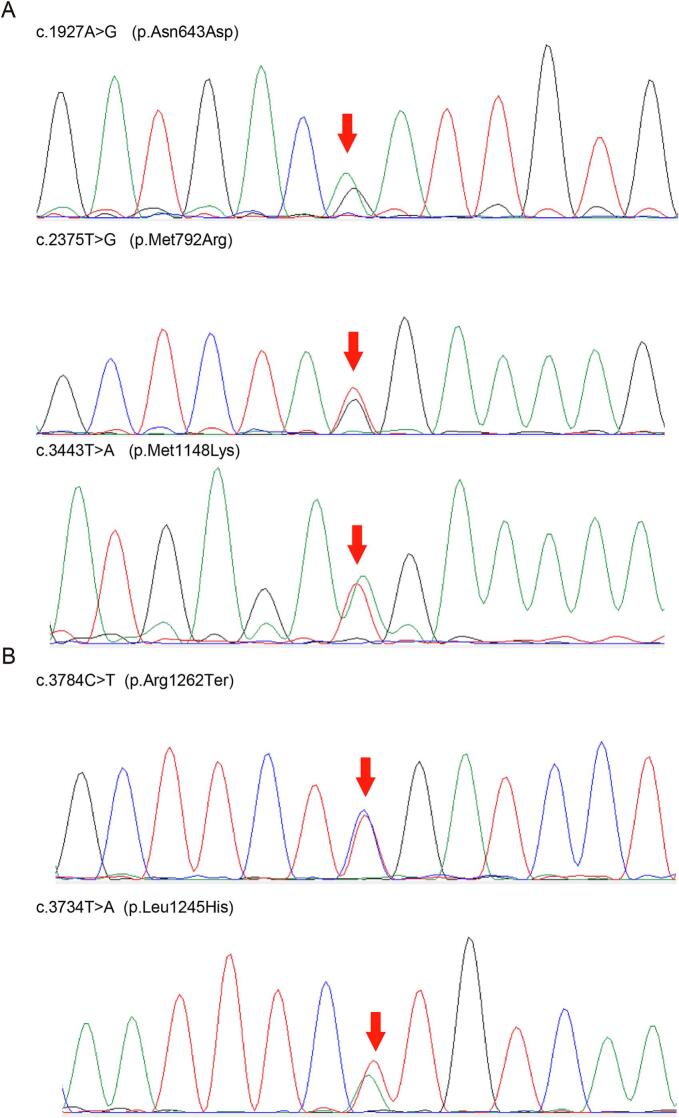
Sanger sequencing of patients with CPS1 variants. A. P1 has three heterozygous variants of c.1927 A > G (p.Asn643Asp), c.2375 T > G (p.Met792Arg) and c.3443 T > A (p.Met1148Lys). B. P2 has two heterozygous variants of c.3784C > T (p.Arg1262Ter), c.3734 T > A (p.Leu1245His). The red arrows refer corresponding variants.

First patient (P1) was the 2nd child to non-consanguineous healthy parents from China, he has a healthy brother. He was hospitalized for fever, poor feeding and poor response on the third day of life and was diagnosed as acute infection at first and went into respiratory failure 7 h later and exhibited encephalopathy, Multiple physiological reflexes disappeared include pupillary light reflex, primitive reflexes, et al. He presented hyperammonemia (>701 μmol/L, reference value 16–60 μmol/L) in his first laboratory tests and the ammonia index rapidly up to >1400 μmol/L. He survived following emergency management which consists of mechanical ventilation, adequate calories with intravenous glucose, liquid infusion and antibiotics. L-arginine and mannitol were administered to lower blood ammonia and high intracranial pressure. Continuous venovenous hemodiafiltration (CVVHDF) treatment were performed in the subsequent 3 days. His ammonia index decreased from >1400 μmol/L, 935 μmol/L, 471 μmol/L to 178 μmol/L. Result of tandem mass spectra suggested urea cycle defects of P1 (elevated glutamine, Table 1). Considering the poor prognosis, his parents gave up his treatment and he developed into death. Patient 1 shows a significant increase of glutamine, 4325.3 μmol/L (reference, 5.0–1150.0 μmol/L), next-generation sequencing and sanger sequencing confirmed 3 heterozygous missense variants in CPS1, c.1927 A > G (p.Asn643Asp), c.2375 T > G (p.Met792Arg) and c.3443 T > A (p.Met1148Lys) (Fig. 1A).

Patient 2 (P2) was the 2nd child to non-consanguineous healthy parents from China, he has a healthy brother. He was hospitalized due to hypothermia and drowsiness on the second day of life. This patient received immediate treatment include liquid infusion and antibiotics, meanwhile, oral feeding was forbidden. He developed seizures during hospitalization and was treated with chloral hydrate promptly. Continuous venovenous hemodiafiltration (CVVHDF) treatment was administered immediately with L-arginine, l-carnitine, vitamin B1, B2, C, coenzyme Q10 and lactulose after hyperammonemia conformed (>700 μmol/L, reference value 16–60 μmol/L). His ammonia index remained >700 μmol/L, reflecting the limited effectiveness of his treatment. Result of tandem mass spectra suggested urea cycle defects (decreased citrulline, elevated ORN/CIT Table 1). His parents gave up his treatment and he developed into death 5 days after birth. He shows a decrease blood index of citrulline, 1.58 μmol/L (reference, 6.00–50.00 μmol/L) and an increase index of ornithine/citrulline, 34.43 (reference, 1.60–25.00), next-generation sequencing and sanger sequencing confirmed 1 heterozygous nonsense variant c.3784C > T (p.Arg1262Ter) and 1 missense variants c.3734 T > A (p.Leu1245His) in CPS1 (Fig. 1B). These variants were confirmed in patients by Sanger sequencing; however, parental testing in these two families was not conducted, and no other variants associated with UCDs or other amino acid metabolism disorders were identified in these two patients.

In summary, NGS found 2 or 3 heterozygous variants of the CPS1 gene in each patient, individually, c.1927 A > G (p.Asn643Asp), c.2375 T > G (p.Met792Arg) and c.3443 T > A (p.Met1148Lys) in P1 and c.3784C > T (p.Arg1262Ter), c.3734 T > A (p.Leu1245His) in P2. The results confirmed by sanger sequencing are helpful in final diagnosis. All variants have been submitted to both LOVD database and ClinVar database, the variants will be accessed using the following URL: https://databases.lovd.nl/shared/genes/CPS1 and https://www.ncbi.nlm.nih.gov/clinvar.

### 3.2 Structural analysis of patients with CPS1 missense variants

**Fig. 2:**
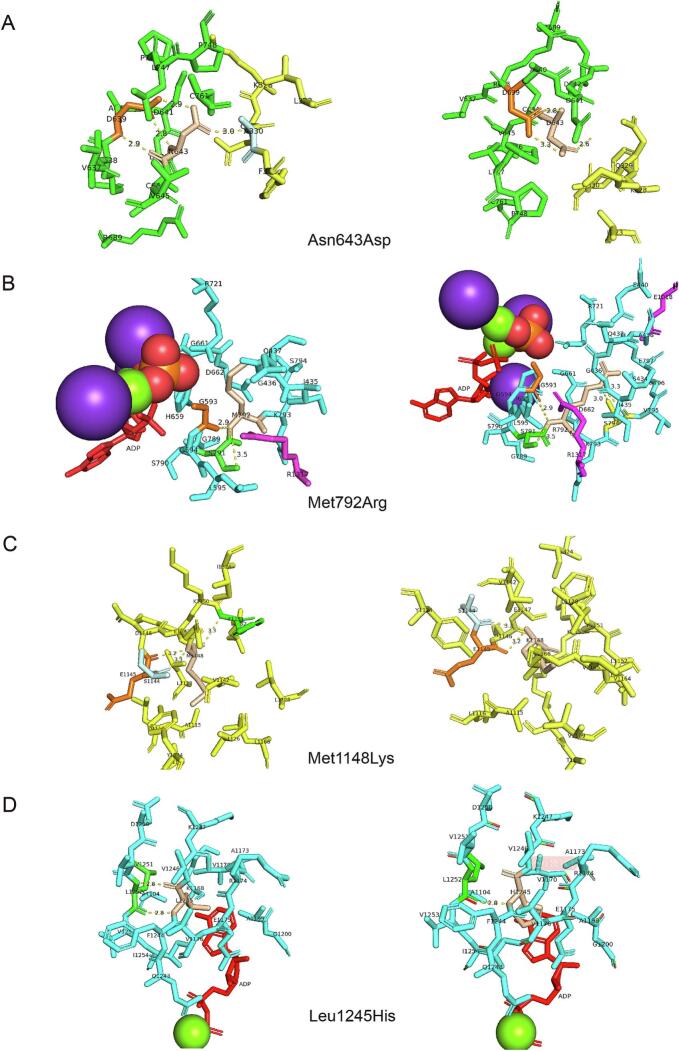
The non-covalent interactions between wild-type and mutant structure, non-covalent bonds are represented by dotted lines. The ADP molecules are colored in red, magnesium ion is shown as a green spheres and the potassium ion is shown as a violet sphere. A. Asn643Asp result in non-covalent interaction changes and might lead to a reduction in the ability of the enzyme in ligand binding in process of bicarbonate phosphorylation. S2 and L1 domain are colored in yellow and green respectively. B. Met792Arg result in in non-covalent interaction changes and might lead to a reduction in the ability of the enzyme in ligand binding in process of bicarbonate phosphorylation. L1 and L3 domain are colored in cyan and violet respectively. C. Met1148Lys result in non-covalent interaction changes might lead to a reduction in the ability of the enzyme in ligand binding in process of carbamate phosphorylation. L3 domain are colored in yellow. D. Leu1245His result in non-covalent interaction changes might lead to a reduction in the ability of the enzyme in ligand binding in process of carbamate phosphorylation. L3 domain are colored in cyan.

Given that nucleotide changes lead to premature termination which not occurring in the 3′ most exon or the 3′-most 50 bp of the penultimate exon in most known genes include CPS1 usually result in nonsense-mediated decay (NMD) rather than structural changes [14,48], only missense variants are structurally analyzed in this study as the nonsense variant is present in exon32 out of 38 exons. 4 missense variants in 2 patients are analyzed using multiple predictors include the difference in thermodynamic stability, flexibility and dynamics of protein structures, which are basic elements of protein function. The 4 missense variants in our patients exhibited a tendency to unstable status as shown in Table 2, represented by the free energy change. For the flexible classification, c.3734 T > A (p.Leu1245His) changed from rigid to intermediate (Table 3) while flexibility of other missense variants remained. The non-covalent interaction changes between the mutated sites and the adjacent residues in the wild-type and mutant structures are well defined in 3D viewer provided by PyMOL (Fig. 2).

**Table 2:** Free energy changes of CPS1 missense variants.

variant	ΔΔG(kcal/mol)			
	PremPS⁎†	mCSM†	DynaMut2†	Summary
Asn643Asp	1.3	−2.008	−1.02	Destabilizing
Met792Arg	2.23	−0.389	−0.6	Destabilizing
Met1148Lys	2.4	−1.833	−0.96	Destabilizing
Leu1245His	1.65	−2.587	−0.79	Destabilizing

⁎ Positive and negative sign corresponds to destabilizing and stabilizing variants.

† Positive and negative sign corresponds to stabilizing and destabilizing variants.

**Table 3:** Flexibility changes of CPS1 missense variants.

Variant	PredyFlexy					
	Class(WT)	B-factor (WT)	RMSF (WT)	Class(Mut)	B-factor (Mut)	RMSF (Mut)
Asn643Asp	intermediate	0.016	0.084	intermediate	−0.145	−0.010
Met792Arg	flexible	0.881	0.788	flexible	0.881	0.788
Met1148Lys	intermediate	−0.177	0.144	intermediate	−0.067	0.014
Leu1245His	rigid	−0.252	−0.017	intermediate	−0.206	−0.041

There are two active centers in charge of bicarbonate phosphorylation and carbamate phosphorylation separately in CPS1. The annotations of HOPE indicates that the variant c.1927 A > G (p.Asn643Asp) is located within the domain responsible for binding other molecules and in contact with residues in a domain that is also important for binding, the substitution might disturb the interaction between these two domains and as such affect the function of the protein. PyMOL shows non-covalent interactions disruption between amino acids 643 and 639 in L1 domain and between amino acids 330 in S2 domain. L1 domain is in charge of bicarbonate phosphorylation but the function of S2 domain has not be characterized to date. The amino acids 643 loci in L1 domain but not close to ATP/ADP binding site, and build up non-covalent interaction with S2 domain. We suppose amino acid change leads to a destabilization consequence of CPS1 protein rather than ATP/ADP binding failure. The residue c.2375 T > G (p.Met792Arg) is buried in the core of carbamoyl phosphate synthase, large subunit. The differences between the wild-type and mutant residue might disturb the core structure. The No.792 residue loci in L1 domain, T-loop region according to its' crystal structure, which catalyzes the phosphorylation of bicarbonate by one ATP molecule each time [49]. This substitution build up new non-covalent interactions between amino acids 792 and 794, which might lead to a reduction in the ability of the enzyme in ATP/ADP binding, result in low production of carbamate intermediate and interfering the smoothly catalyzed reaction. The other 2 substitutions, c.3443 T > A (p.Met1148Lys) and c.3734 T > A (p.Leu1245His) are located in the same domain, the ATP-grasp fold domain in L3, which phosphorylate carbamate by one ATP molecule to produce carbamoyl phosphate. As is shown in schematic structure, non-covalent interaction between amino acids 1148 and 1151 has been disrupted, though Met1148 could not be traced in 5DOU, but it is close to one reaction center of ATP/ADP catalytic centers, and is likely interrupt the ATP binding and metal ion binding process which perform in this domain. Amino acids 1245 has been described as ADP binding site previously, the substitution will result in interruption of ATP/ADP binding. Both phosphorylation steps occurring at two separate active centers of CPS1 are disrupted due to amino acid substitutions, result in disruptive change in well-organized catalytic flow.

### 3.3 Variants pathogenicity interpretation of CPS1D patients under ACMG/AMP-ClinGen guidelines

Table 4 summarized the main genetic characteristics and in silico investigations of missense variants found in 2 CPS1D patients. The 2 unreported variants c.1927 A > G (p.Asn643Asp) and c.2375 T > G (p.Met792Arg), and 1 reported variant c.3443 T > A (p.Met1148Lys) [49] are identified in P1, then all 3 variants are classified as VUS based on the Standards and Guidelines for the Interpretation of Sequence Variants by the ACMG/AMP-ClinGen (Table 5). The missense variant c.3734 T > A (p.Leu1245His) and nonsense variant c.3784C > T (p.Arg1262Ter) found in P2 have also been reported in cases [33,40,42,51]. According to the guidelines, c.3734 T > A (p.Leu1245His) is classified as likely pathogenic (LP) while c.3784C > T (p.Arg1262Ter), pathogenic (P). All the admissible criteria are listed in Table 5.

**Table 4:** Genotypic features and in silico investigations of CPS1 missense variants and Comparative evaluation of pathogenicity interpretation of CPS1 variants before and after PP3/BP4 criteria updating.

Patient	Exon	Variant (NM_001875)	CPS1 Domain⁎	gnomAD Exomes frequency	REVEL score	Criteria(before)	ACMG (before)	Criteria(after)	ACMG (after)
P1	Exon17	c.1927 A > G (p.Asn643Asp)	BPSD	–	0.947	PM1, PM2_Supporting, PP3, PP4	VUS	PM1, PM2_Supporting, PP3_ Moderate, PP4	LP
	Exon19	c.2375 T > G (p.Met792Arg)	BPSD	–	0.9409	PM2_Supporting, PP3, PP4	VUS	PM2_Supporting, PP3_ Moderate, PP4	VUS
	Exon28	c.3443 T > A (p.Met1148Lys)	CPSD	–	0.921	PM2_Supporting, PP3, PP4	VUS	PM2_Supporting, PP3, PP4	VUS
P2	Exon31	c.3734 T > A (p.Leu1245His)	CPSD	–	0.944	PM1, PM2_Supporting, PM3, PP3, PP4	LP	PM1, PM2_Supporting, PM3, PP3_ Moderate, PP4	LP
	Exon32	c.3784C > T (p.Arg1262Ter)	CPSD	0.00000752	–	PVS1, PM2_Supporting, PM3, PP4	P	PVS1, PM2_Supporting, PM3, PP4	P

⁎ Distribution of variants in CPS1 domain corresponds to previous report [49]. BPSD, bicarbonate phosphorylation; CPSD, carbamate phosphorylation.

**Table 5:** Pathogenicity interpretation of CPS1 variants.

Patient	Variant (NM_001875)	Criteria	ACMG	Reference
P1	c.1927 A > G (p.Asn643Asp)	PM1, PM2_Supporting, PP3, PP4	VUS	Novel
	c.2375 T > G (p.Met792Arg)	PM2_Supporting, PP3, PP4	VUS	Novel
	c.3443 T > A (p.Met1148Lys)	PM2_Supporting,PP3, PP4	VUS	[50]
P2	c.3734 T > A (p.Leu1245His)	PM1, PM2_Supporting, PM3, PP3, PP4	LP	[51]
	c.3784C > T (p.Arg1262Ter)	PVS1, PM2_Supporting, PM3, PP4	P	[33,40,42,51]

### 3.4 Comparative evaluation of pathogenicity interpretation of CPS1 missense variants in patients and ClinVar database

To decoding the importance of in silico predictions in pathogenicity interpretation, we made a comparation assessment of variants in our patients firstly. The c.1927 A > G (p.Asn643Asp) VUS variant upgraded to LP, other reported variants maintain their pathogenicity classification (Table 4).

**Fig. 3:**
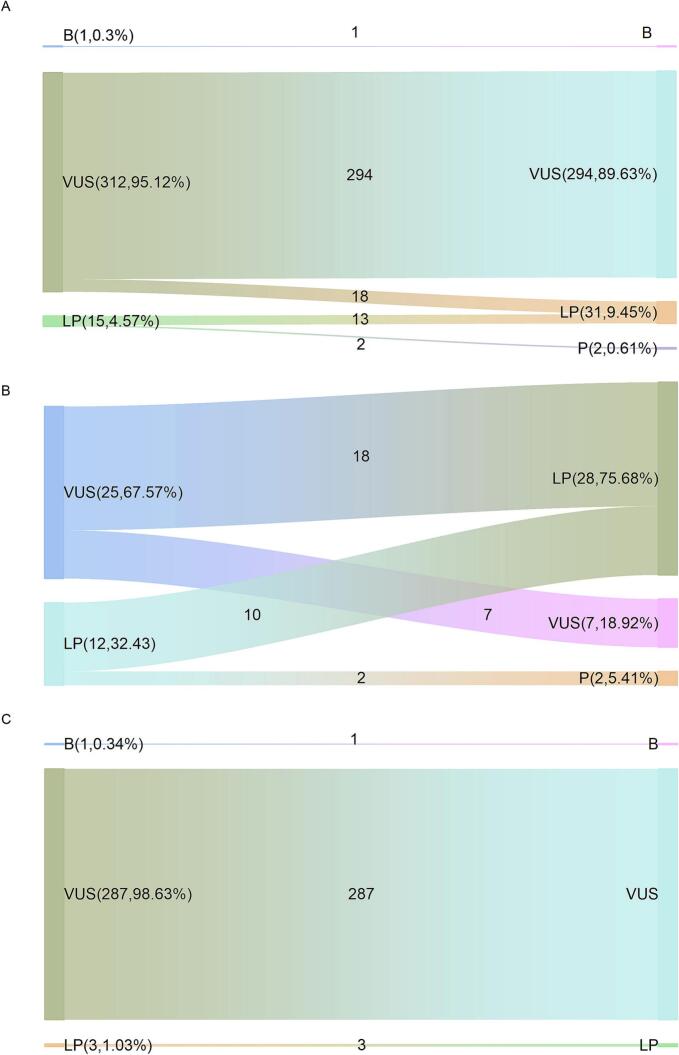
Distribution and flow changes of CPS1 pathogenicity classification in ClinVar. A. Left: 95.12 % (312 variants) are classified as VUS, 4.57 % (15 variants) are classified as LP and 0.3 % (1 variant) are classified as B in ClinVar Database before PP3 criterion application; Right: 89.63 % (294 variants) are classified as VUS, 9.45 % (31 variants) are classified as LP and 0.61 % (2 variants) are classified as P and 0.3 % (1 variant) are classified as B in ClinVar Database after PP3 criterion application. B. Reported variants group; Left: 67.57 % (25 variants) are classified as VUS and 32.43 % (12 variants) are classified as LP before PP3 criterion application; Right: 18.92 % (7 variants) are classified as VUS, 75.68 % (28 variants) are classified as LP and 5.41 % (2 variants) are classified as P after PP3 criterion application. C. Unreported variants group; Left: 98.63 % (287 variants) are classified as VUS, 1.03 % (3 variants) are classified as LP and 0.34 % (1 variants) are classified as B before PP3 criterion application; Right: 98.63 % (287 variants) are classified as VUS, 1.03 % (3 variants) are classified as LP and 0.34 % (1 variants) are classified as B after PP3 criterion application.

Considering that the pathogenicity assessment conclusions of a handful of variants are insufficient to demonstrate effects of these criteria updating, a larger number of variants should be taken into consideration. To making the further understanding of this updating, we performed the comparation assessment of CPS1 missense variants in ClinVar database. A total of 328 missense variants in CPS1 are included in this study, the pathogenicity assertion, corresponding criteria and references are listed in Table S1. 95.12 % of these variants (312 variants) are classified as VUS, 4.57 % (15 variants) as LP and 0.3 % (1 variant) as B, while the distribution listed as 89.63 % (294 variants) VUS, 9.45 % (31 variants) LP, 0.61 % (2 variants) P and 0.3 % (1 variant) as B when the new guideline is applied (Fig. 3A).

The variants were further divided into two groups depending on whether they have been reported in the literature, the group of reported variants in cases and the group of unreported variants to uncover the different impact of this application separately. There are 25 VUS and 12 LP variants in 37 reported variants, which accounted for 67.57 % and 32.43 % before PP3/BP4 criterion application, the data layout transformed to 18.92 % (7 variants) VUS, 75.68 % (28 variants) LP and 5.41 % (2 variants) P after the practical application for PP3/BP4 evidence criteria (Fig. 3B). The flow change shows18 VUS variants are upgrading to LP and 2 LP variants to P, 54 % totally. In unreported variants group, almost all the variants are assessed as VUS variants expect the ones which are associated with PM5 (Novel missense change at an amino acid residue where a different missense change determined to be pathogenic has been seen before). The rule of limiting the sum of the evidence strength of PP3 and PM1 to Strong allows most unreported variations keep their pathogenicity classification at VUS level, resulting in the distribution of ∼98.63 % (287 variants) VUS and ∼ 1.03 % (3 variants) LP (Fig. 3C). The distribution of pathogenicity classification remains same in this group after application for PP3/BP4. It appears that the updating criteria of PP3/BP4 encourages a clearer pathogenicity interpretation while preventing inappropriate classification of variants in the absence of cases.

## 4 Discussion

Inherited metabolic diseases include CPS1D are often early onset, rapid deterioration and high mortality which can be obviously improved by early diagnosis and treatment [52]. CPS1 is the enzyme in the process of ammonia detoxification of the urea cycle at proximal step of catalytic. Dysfunctional CPS1 are characterized by hyperammonemia, increased glutamine concentrations and hypocitrullinemia clinically, significantly high glutamate and glutamine levels in patients with CPS1D would be considered a major contributor to hyperammonemia metabolism [53]. Though glutamate and glutamine levels are not required diagnostic criteria that included in the guideline, they are still sensitive as a predictor of clinical severity of hyperammonemic crises [54,55]. The treatments are suggested to perform as soon as possible to reduce those severe prognoses such as neonatal death or irreversible encephalopathy cause by exposure of hyperammonemia [12]. Though variant detection is the preferred method for diagnosis when metabolite profiles are not informative [12], sensitive indicators are helpful in screening as well as diagnosis at first step. We defined the cut-off value (5.0–1150.0 μmol/L) of glutamine, based on the data of 100,000 newborns according to the Chinese Expert Consensus on the methodology for establishing cut-off values of screening indicators for neonatal inherited metabolic diseases [56], and makes glutamine as another sensitive predictor of UCDs in MSMS, rather than fluctuation of citrulline only in this study. Our final diagnosis based on NGS proved one patient who did not experience citrulline level change carries genetic variants of CPS1. In a word, glutamine could be an alternative actively supplementing reminder parameter when positive results achieved in suspected genetic metabolic disorders in diagnosing UCDs in order to perform early intervention.

Early diagnosis for patients with non-characteristic changes is of great importance, advances in sequencing technology have made it possible to make diagnosis closer to the asymptomatic period once interpretation of variants are clear enough. The pathogenicity classification of variants is of great significance in clinical diagnosis for patients with suspected genetic diseases and the following genetic counseling for families. It is admitted in the original guidelines that these classification approaches are more stringent than laboratories have applied ever, and will result in a larger proportion of variants being categorized as VUS [13]. With the decrease of cost and optimization of analytical methods, NGS is widely used in rare disease diagnosis day by day. However, VUS variants are discovered at a rate that outpaces current ability to classify them with databases of previous cases, experimental evaluation, and computational predictors [57]. De novo variants are easily found in patients with dominant inherited disease without regard to X-linked (XL) pattern and incomplete penetrance. Pathogenicity classification of missense variants in autosomal dominant (AD) disorders are easier compare to AR disorders due to higher weight of de novo variants in the assessment process based on confirmed parental relationships. The previous cases and experimental evaluation are two effectively but not readily available approaches for pathogenicity interpretation. The status illustrates the importance of increasing weight of in silico predictions. Historically, variety of algorithms focus on different aspects have been developed in the past two decades in order to perfect computational predictors [58]. While the defined weight of computational predictions has not been changed since then, clinic pathogenicity of variants did not been affected ever. The new guideline for PP3/BP4 differ different weights based on the score run by in silico predictors respectively, result of reduction in VUS can be expected in NGS. In our study, 54 % reported variants are upgraded from VUS to LP through the application of new PP3/BP4 criteria. Further analysis of assessment illustrates PM3, the phase information of cases, and reappraisal in PP3/BP4 are important elements in pathogenicity assessment (Table S1).

Interpretation of 2 or more variants in cis are more complex and unpredictable. The separated interpretation of variants in cis may not reflect the consequences accurately. For example, the c.-119_-116delGTCA and other 4 variants (c.940 A > G, c.378-27G > C, c.508-24G > A, c.507 + 62G > A) that associated with Duarte Variant Galactosemia, each of these variants are considered as benign separately while the combination of cis-forms variants shows higher frequency in Duarte Variant Galactosemia patients than controls [59]. *cis*-Variants need to be evaluated with more caution in variant interpretation. Patient 1 was found to have three variants: c.1927 A > G (p.Asn643Asp), c.2375 T > G (p.Met792Arg), and c.3443 T > A (p.Met1148Lys). Among these, only c.3443 T > A (p.Met1148Lys) has been previously reported. The c.1927 A > G (p.Asn643Asp) variant is located in a mutational hotspot within the L1 domain, while c.2375 T > G (p.Met792Arg) is situated near one of the ATP/ADP catalytic reaction centers in the L3 domain, as illustrated in the schematic structure. Each of these three variants is likely to be deleterious on its own, and the combination of different variants introduces unpredictability in terms of their cumulative effects.

Though more practical clinic data in different genes are still in need, our result shows an optimistic vision of future for VUS interpretation for doctors, genetic counselors and family members of cases.

In conclusion, this study identified novel *CPS1* variants and analyzed the impact of *CPS1* missense variants associate with updated PP3/BP4 criteria, proved that this improvement is beneficial for practical clinic.

The following are the supplementary data related to this article.

Supplementary Table S1 in the Supplementary Material for 130 Genes list of Inherited Metabolic Diseases. Supplementary Table S1

Supplementary Table S2 in the Supplementary Material for comparation assessment of CPS1 missense variants in ClinVar database. Supplementary Table S2

## Institutional review board statement

The study was conducted according to the guidelines of the Declaration of Helsinki, and approved by Ethics Review Committee of Shanghai Children's Hospital, the Affiliated Children's Hospital of Shanghai Jiao Tong University School of Medicine in China (protocol code 2019R071 and date of approval: 26 March 2020).

## Informed consent statement

Informed consent was obtained from all subjects involved in the study.

## Funding

This work was supported by the 10.13039/501100012166National Key Research and Development Program of China, Grant/Award Number: 2019YFA0801402, 2024YFC2707002; the 10.13039/501100001809National Natural Science Foundation of China, Grant/Award Number: 82271890; Shanghai Key Clinical Specialty Project, Grant/Award Number: shslczdzk05705; Shanghai Top Priority Key Discipline Project, Grant/Award Number: 2017ZZ02019; Innovative Research Team of High-Level Local Universities in Shanghai, Grant/Award Number: SHSMU-ZDCX20212200; the Macau Science and Technology Development Fund (FDCT) (0092/2022/A2 and 003/2022/ALC).

## CRediT authorship contribution statement

**Fei Li:** Writing – review & editing, Writing – original draft, Methodology, Investigation, Formal analysis, Data curation, Conceptualization. **Qin Cai:** Writing – review & editing, Methodology, Investigation, Data curation, Conceptualization. **Wei Ji:** Methodology, Investigation, Formal analysis, Data curation. **Miao Xu:** Methodology, Investigation. **Guoli Tian:** Writing – review & editing, Methodology. **Fanyi Zeng:** Writing – review & editing, Funding acquisition.

## Declaration of competing interest

The authors declare no conflict of interest.

## Data Availability

Data will be made available on request.
